# Laparoscopic Treatment of an Incarcerated Meckel's Diverticulum in a Femoral Hernia

**DOI:** 10.1155/2019/3140706

**Published:** 2019-08-08

**Authors:** Christoph Paasch, Gianluca De Santo, Peter Look, Katherina Boettge, Michael Hünerbein

**Affiliations:** ^1^Department of General, Visceral and Cancer Surgery, Helios Klinikum Berlin-Buch, Schwanebecker Chaussee 50, 13125 Berlin, Germany; ^2^No Insurance Surgery (NIS), 9121 W Russell Rd, Las Vegas, NV 89148, USA

## Abstract

Meckel's diverticulum (MD) is the persistence of the omphalomesenteric duct. It is usually asymptomatic but may present with bleeding, infections, and intestinal obstruction. It also may be a content of a hernia sac, a so-called Littre hernia. Herein, we will present the case of a 75-year-old female, who suffered from a painful swelling of the right inguinal region. Ultrasound imaging detected an inguinal hernia with incarcerated blind ending small bowel. Immediately, a laparoscopy was conducted. We diagnosed a right femoral hernia with an incarcerated MD. A TAPP (transabdominal preperitoneal) procedure was performed and the MD tangential stapled. Due to an uneventful postoperative course, the patient left the hospital after two days. An incarceration of a MD in a femoral hernia is rare. Tangential resection of the MD with simultaneous hernia repair in a TAPP technique seems to be a sufficient approach, when it is conducted by an experienced surgeon.

## 1. Introduction

Meckel diverticulum (MD) is the persistence of the omphalomesenteric duct through which the midgut communicates with the umbilical vesicle until the fifth week [1]. It is the most common anomaly of the gastrointestinal tract. Its embryological origin was first described in 1809 by the German anatomist Meckel (1781 to 1833) [1]. MD can be diagnosed at the antimesenteric border of the ileum, and it is usually located 30 to 90 cm from the ileocecal valve [1]. MD is usually asymptomatic but may present with bleeding, infections, intestinal obstruction, and ectopic hormone secretion. Moreover, MD can be a content of a hernia sac, a so-called Littre hernia [2]. In these cases, it may undergo incarceration or strangulation, necrosis, and perforation. Incarceration of MD in a femoral hernia has been rarely described in the literature especially with a sufficient colored intraoperative imaging.

## 2. Case Presentation

Herein, we present the case of a 75-year-old female. She was referred to our hospital because of an increasing inguinal pain for 8 days. Her medical history consisted of multimodal treatment of breast cancer in 2010. She moreover underwent open inguinal hernia repair on both sides in her childhood. During the clinical examination at our emergency room, a painful swelling of the right inguinal region was revealed. The patient did not have a clinical peritonitis sign. Defecation and urination were sufficient. A laboratory test revealed normal concentrations of the C-reactive protein and leucocytes. We performed an ultrasound imaging and detected an inguinal hernia with an obviously blind ending small bowl in the hernia sac.

**Figure 1:**
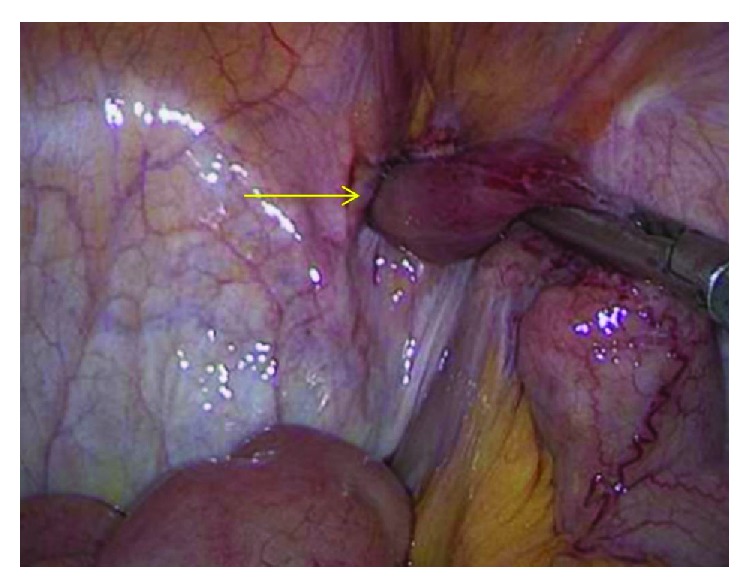
The incarcerated insufficient blood-supplied Meckel's diverticulum is marked by the yellow arrow.

**Figure 2:**
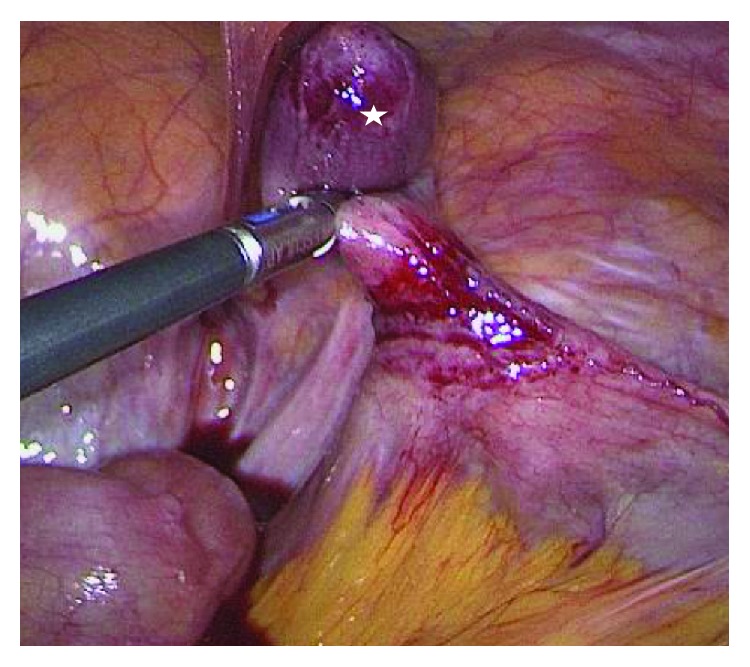
The white star indicates the released Meckel's diverticulum from the hernia.

Immediately, an exploratory laparoscopy was conducted. A 10 mm metal trocar was inserted supraumbilically, and a pneumoperitoneum was created with a pressure of 15 mm Hg. A 12 mm trocar into the right midabdomen and a 5 mm trocar into the left mid-abdomen were inserted. We then diagnosed a right femoral hernia with an incarcerated MD (Figure 1). After the removal of the hernia sac content, a hernia repair in a TAPP technique with the placement of a 15 × 10 cm Parietene mesh (Covidien©) was conducted. The hernia gap sized 1.5 cm. The small bowel had sufficient blood supply without any sign of ischemia, but the MD had insufficient blood supply and was tangentially removed using a stapler (Figures 1 and 2).

The postoperative course was uneventful. The patient was discharged two days after surgery. The histopathological examination of the resected small bowel segment confirmed the diagnosis of an insufficient blood supplied MD. Neither malignancy nor mucosa dystopia was detected.

## 3. Discussion

A protrusion of a MD through a potential abdominal opening was first described by the French surgeon Alexis de Littre in 1700 [2]. The usual sites of these so-called Littre hernias are inguinal (50%), umbilical (20%), and femoral (20%) [3]. A Littre hernia occurs in less than 1% of patients with a MD [3]. An MD incarceration in femoral hernia like in the case report at hand is very rare. The first description was in 1924 by the British surgeon Littler [3, 4]. Until today, only a very small amount of publications are available. Reviewing the literature on this topic, Racy and Ramesh published that in out of 680 patients who suffered from an incarcerated femoral hernia, only one patient had a Littre hernia [3]. Moreover, femoral hernias rarely occur and represent only 4–5% of groin hernias [5]. They are more common on the right side like in the case report at hand probably due to the anatomical position of the sigmoid colon. The bowel may tampon the femoral canal [5].

In terms of the surgical approach, an inguinal incision with a nonmesh hernia repair is often advised in cases of femoral hernia incarceration [3]. In an elective setting to repair these hernias, the TAPP procedure has already been described in the literature as a sufficient and feasible approach with less postoperative pain in comparison to open hernia repair [5]. Like mentioned, we conducted an exploratory laparoscopy with tangential MD resection and hernia repair in the TAPP technique. We favored this approach because laparoscopy may facilitate sufficient evaluation of blood supply and vitality of the bowel. To that, in cases of negative findings, an exploratory laparotomy or inguinal incision may represent an unnecessarily extended surgical approach. Also, Comman et al. and Malling et al. laparoscopically treated a patient who suffered from an incarcerated femoral Littre hernia. They successfully conducted a hernia repair in the TAPP technique with the resection of MD and reported an uneventful postoperative course of their patient [4, 6].

For correct hernia repair, a mesh was placed in our case report only due to the lack of an abdominal infection.

## 4. Conclusion

The incarceration of a Meckel's diverticulum in a femoral hernia is rare. Tangential resection of the MD with simultaneous hernia repair in the TAPP technique seems to be a feasible and sufficient approach, when it is conducted by an experienced surgeon.
